# Carer Café in Taiwan: An Innovative Model Providing Caregiver Support for Older Adults

**DOI:** 10.1093/geroni/igab046.1874

**Published:** 2021-12-17

**Authors:** Su-I Hou, Tsuann Kuo

**Affiliations:** 1 School of Global Health Management & Informatics, University of Central Florida, University of Central Florida, Florida, United States; 2 Chung Shan Medical University, Taichung, Taichung, Taiwan (Republic of China)

## Abstract

Physical places and environments play critical roles in shaping how people interact. This paper introduces an innovative Carer Café model in Taiwan with social infrastructure support and services for family caregivers caring for older adults. Carer cafés are community-based initiatives of Taiwan Association of Family Caregivers (TAFC), aiming to help carers recognizing their own needs and increase awareness of long-term care resources available. Through partnerships with local coffee shops, respite-focused services are provided. The “free coffee for two” campaign encourages family members and friends taking a caregiver out for a “respite coffee”. Carer Café has also become an important “third place” hosting respite programs and the “a shop within a shop” style creating designated space to build a sense of community. Pilot survey (n=375) showed 77% perceived reduced stress, 83% appreciated services provided, and 250% increased referrals within one year. Implications on impact and future opportunities will be discussed.

